# Investigation of the Na–Ga Phase Diagram

**DOI:** 10.1021/acsomega.5c11531

**Published:** 2026-01-05

**Authors:** Chia-Chi Yu, Marcus Schmidt, Michael Baitinger, Yuri Grin

**Affiliations:** 28270 Max-Planck-Institut für Chemische Physik fester Stoffe, Nöthnitzer Straße 40, 01187 Dresden, Germany

## Abstract

The Na–Ga phase diagram was reinvestigated by heat-flux differential scanning calorimetry (HF-DSC) and powder X-ray diffraction (PXRD). The most sodium-rich phase, Na_22_Ga_39_, melts congruently (549(2) °C), while Na_7_Ga_13_ (545(2) °C), Na_2_Ga_7_ (501(2) °C), and NaGa_4_ (495(2) °C) decompose peritectically. In the sodium-rich region, a monotectic reaction between Na_22_Ga_39_ and melt occurs at 495(2) °C. The critical temperature of the liquid two-phase region was estimated to 523(2) °C. The subtle differences in the decomposition temperatures of neighboring phases were resolved by differential thermal analysis using a mutual sample reference method.

## Graphical abstract

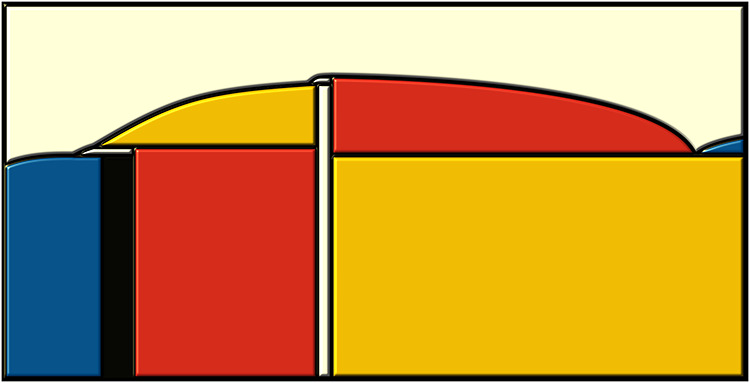

## 1 Introduction

Comprehensive reviews of the binary Na–Ga system have been provided by Itami et al.,^1^ Pelton et al.^2^ and Okamoto.^3^ So far, four intermetallic phases have been identified: NaGa_4_ (20 atom % Na),^4–6^ Na_2_Ga_7_ (22 atom % Na),^7^ Na_7_Ga_13_ (35 atom % Na),^5^ and Na_22_Ga_39_ (36 atom % Na).^8,9^ The previous studies reported conflicting conclusions on the phase equilibria. Regarding the melting behavior of NaGa_4_, Pelton et al. suggested congruent melting, whereas Okamoto described a peritectic reaction. The coexistence of the phases Na_7_Ga_13_ and Na_22_Ga_39_ is also controversial. While Pelton and Okamoto considered only Na_22_Ga_39_ as an equilibrium phase, Itami et al. reported both phases. The phase Na_2_Ga_7_ was identified only recently^7^ and therefore not included in the earlier assessments. Furthermore, the phase equilibria in the sodium-rich part of the phase diagram remain unclear. Both Pelton and Okamoto reported a miscibility gap in the liquid state with a monotectic reaction, but they proposed significantly different critical temperatures for this gap. In contrast, Itami et al. interpreted the isothermal line in the sodium-rich region as a peritectic reaction rather than a monotectic one. Regarding the mutual solid solutions Na­[Ga] and Ga­[Na], the literature agrees that they are negligible and that both form eutectic mixtures with their respective neighboring phases.^10,11^ However, the eutectic melting temperatures are too close to those of the pure elements to be clearly distinguished. In this work, the Na–Ga phase diagram was reinvestigated using HF-DSC and PXRD measurements to resolve the aforementioned discrepancies and to include the equilibrium phase Na_2_Ga_7_.

## 2 Experimental Section

### 2.1 Preparation

All samples were prepared from stoichiometric mixtures of the elements in an argon- filled glovebox (Na, Chempur, 99.9%; Ga, Chempur, 5N). The annealing conditions and crucible material varied depending on the chemical composition. Elemental Na was fixed on a needle, placed inside the respective crucible without touching the side walls, and released from the needle by heating the open crucible to 120 °C on a hot plate. Elemental Ga was solidified on a Peltier cooler, crushed at −40 °C and added to the crucible.

### 2.2 Ga-Rich Samples (up to 8 Atom % Na)

The starting materials were weighed into niobium crucibles suitable for the HF-DSC apparatus (*d* = 5 mm, *h* = 10 mm) and sealed by arc welding under an inert atmosphere. The sealed crucibles were heated to ≈750 °C for 1 min in an induction furnace to homogenize the mixture. Finally, the samples were allowed to rest for 1 day in the glovebox at 25 °C. Before the HF-DSC measurement, the sample were cooled on a Peltier cooler to make sure that the melting of Ga can be detected in the DSC experiments.

### 2.3 Compounds (NaGa_4_, Na_2_Ga_7_, Na_7_Ga_13_, Na_22_Ga_39_)

Approximately 1 g of the stoichiometric mixtures of the elements were sealed in tantalum ampules (*d* = 10 mm, *h* = 40 mm) by arc welding. The ampules were homogenized in an induction coil for 1 min at 1000 °C and subsequently cooled by removing them from the coil. Different parts of the specimens were then annealed for 7 days at 200, 350, and 400 °C and quenched in water. All compounds were obtained as single-phase materials according to PXRD and were found to be sensitive to air and moisture.

### 2.4 Na-Rich Samples (>50 Atom % Na)

Samples in the sodium-rich region were prepared by mixing presynthesized Na_22_Ga_39_ with elemental sodium. The starting materials (*m* ≈ 50 mg) were placed in screwable stainless steel crucibles suitable for the HF-DSC apparatus. For nominal composition Na_*x*_Ga_100–*x*_ (*x* = 60, 75, 80), the samples were annealed at 400 °C for 1 day and cooled to room temperature by removing them from the furnace. Samples with nominal compositions Na_92+*x*_Ga_8–*x*_ were homogenized at 120 °C for 12 h and subsequently kept at ≈25 °C for 1 day.

### 2.5 Heat-Flux Differential Scanning Calorimetry (HF-DSC)

Specimens of 30–50 mg were sealed custom-made niobium crucibles (*d* = 5 mm, *h* = 10 mm, *m* ≈ 600 mg). The crucibles were fabricated by drilling out a niobium rod. After filling, the crucibles were sealed using an arc welder under continuous water cooling of the sample holder to minimize heat transfer to the sample. Subsequently, the sealed ampules were reannealed at their respective synthesis temperatures, counteracting any potential phase changes induced by the welding process. The measurements were conducted under a flowing argon using a heat-flux DSC instrument (Netzsch DSC 404C; DSC sensor type S). The samples were heated from room temperature to 800 °C at rates between 2 and 10 °C/min. For sodium-rich compositions, where welding of short crucibles was not applicable, mechanically sealable crucibles were employed. Two commercial crucibles were used: stainless steel high-pressure capsules with a titanium disc (PerkinElmer), and cylindrical stainless-steel crucibles that were screw sealed (Setaram). Test measurements confirmed that both crucible types remained perfectly sealed when heated with elemental Na up to 1000 °C. All temperatures given in the text refer to the peak onset.

### 2.6 Mutual Reference DSC (MR-DSC)

To discern subtle differences in the thermal effects of phases with similar melting points, mutual reference experiments were performed. In this method, two samples of the phases of interest are measured simultaneously, with one placed in the sample holder and the other in the reference holder of the DSC device. This configuration produces output signals of opposite polarity for each sample holder. While the overlapping signals only allow an estimation of the transition temperatures for the first thermal event, they allow for a relative comparison. The sequence of events is indicated by the initial direction (positive or negative) of the combined signal. To ensure reliability, the measurements were repeated with freshly prepared samples, and the positions of the two phases were swapped. Crucible types and heating rates were identical to those used in standard DSC measurements, and similar sample masses were employed for the phases being compared. Calibration measurements revealed a constant temperature offset of approximately 0.2 °C between the sample and reference sides of the instrument. Consequently, the interpretation by MR-DSC reaches its resolution limit when the difference in thermal events falls within this range, as was observed for sample pairs such as elemental Ga and the assumed eutectic mixtures Ga/NaGa_4_ or Na and Na/Na_22_Ga_39_, respectively.

### 2.7 Powder X-ray Diffraction (PXRD)

All samples were characterized by the Guinier technique using a Huber Image Plate Camera G670 with a germanium monochromator (Cu Kα_1_ radiation; λ = 1.54056 Å). Finely ground specimens were fixed under an argon atmosphere between two polyimide foils (7.5 μm, Kapton, Chemplex), using a film of vacuum grease (Lithelen, Leybold). Data were collected in the 2θ range of 5.0–100° with a step width of 0.005°.

## 3 Results and Discussion

### 3.1 Ga-Rich Samples (up to 20 Atom % Na)

Previous studies of the Na–Ga system consistently assumed a eutectic between NaGa_4_ and α-Ga, with the eutectic temperature and the melting point of α-Ga being too close to be distinguished.^1–3^ This assumption is corroborated by the already minute solubility of Na in liquid Ga at 30 °C, reported to be only 0.003 atom %.^10^ Therefore, any depression of the melting point of Ga due to small solubilities in the solid state would be immeasurably small. Our HF-DSC measurements revealed an endothermic effect upon heating at 30(2) °C (Figures S1a–c), which aligns with the literature value of 29.76 °C for α-Ga.^12^ However, this thermal effect could only be observed in a HF-DSC experiment after forcing the crystallization of gallium before the measurements by cooling the ampules on a Peltier cooler, as the high ambient temperature inside the glovebox otherwise prevents solidification.

### 3.2 Formation of NaGa_4_

A single-phase NaGa_4_ sample (20 atom % Na), as confirmed by PXRD, showed a strong endothermic effect with an onset temperature of 495(2) °C, accompanied by a weak shoulder near 508 °C. This endothermic effect was consistently reproducible for samples in the range of 18–21 atom % Na, indicating an isothermal reaction line (Figure S3a–c). These findings suggest a peritectic formation for NaGa_4_, ruling out the previously discussed congruent melting.^2^ Furthermore, the previously proposed peritectic reaction *L* + Na_22_Ga_39_ → NaGa_4_ ^3^ is not supported by our data. Instead, we conclude that NaGa_4_ forms peritectically at 495(2) °C from its neighboring phase Na_2_Ga_7_ and the melt according to *L* + Na_2_Ga_7_ → NaGa_4_ (Figures 1 and 2a). The refined lattice parameters of NaGa_4_ obtained from samples annealed between 200–450 °C within the composition range of 18–21 atom % Na are constant [for detailed values, see Table 1 in ref 7] confirming that NaGa_4_ is a “line” compound. The liquidus of NaGa_4_ was estimated by HF-DSC measurements on compositions of 9 atom % Na (≈440 °C), 10 atom % Na (≈460 °C), 11 atom % Na (≈480 °C) (Figures S2). The previous interpretation of NaGa_4_ as a congruently melting phase can be rationalized by kinetics: upon cooling a melt of the nominal composition NaGa_4_, the nucleation of the peritectically required primary phase Na_2_Ga_7_ (see below) is kinetically inhibited.^7^ This inhibition leads to the direct crystallization of NaGa_4_, bypassing the equilibrium reaction pathway and mimicking the behavior of a congruently melting compound.

**Figure 1:**
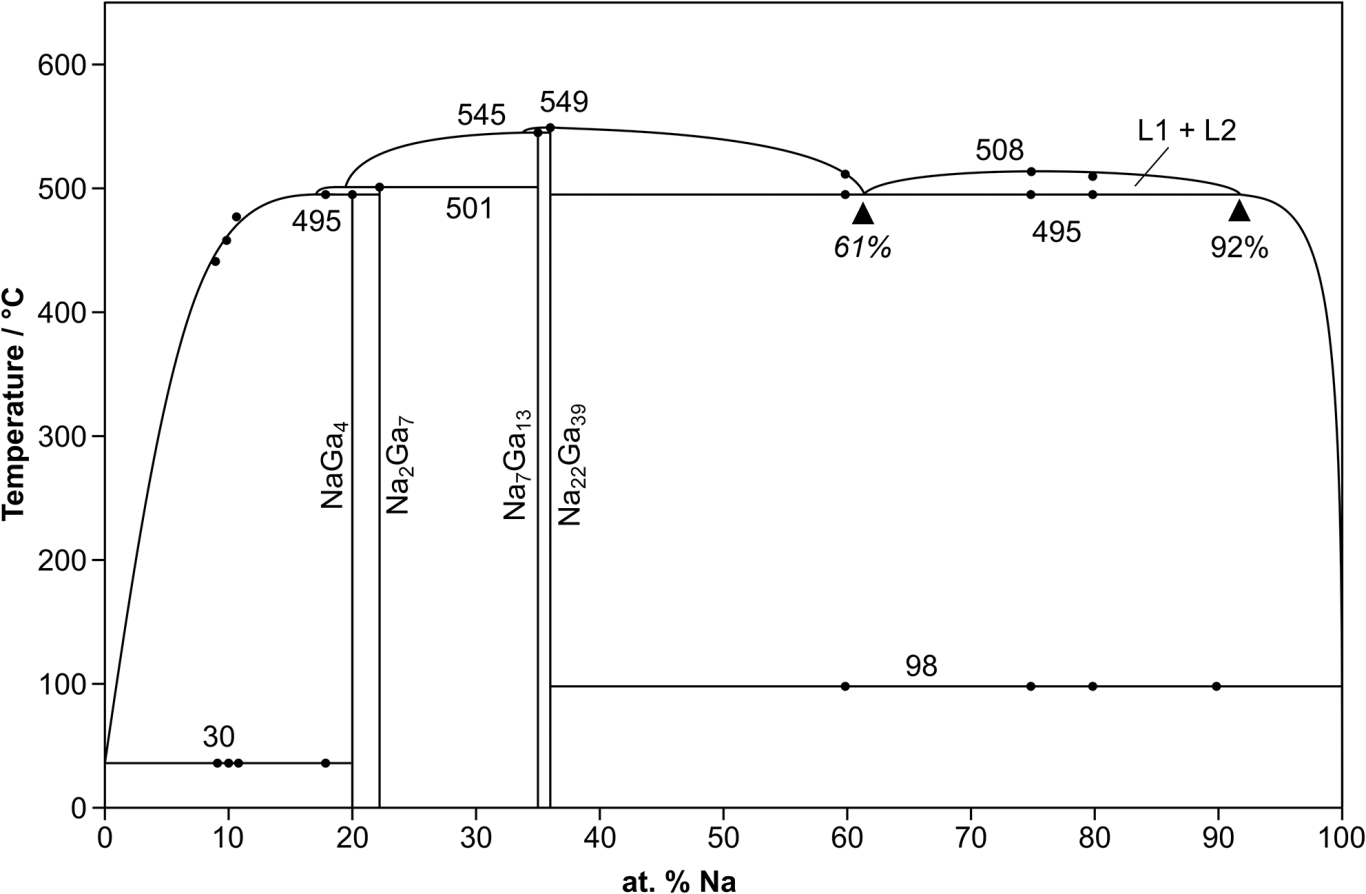
Reinvestigated Na/Ga phase diagram. Experimental data points are marked with black dots; the liquidus curves are estimated.

**Figure 2:**
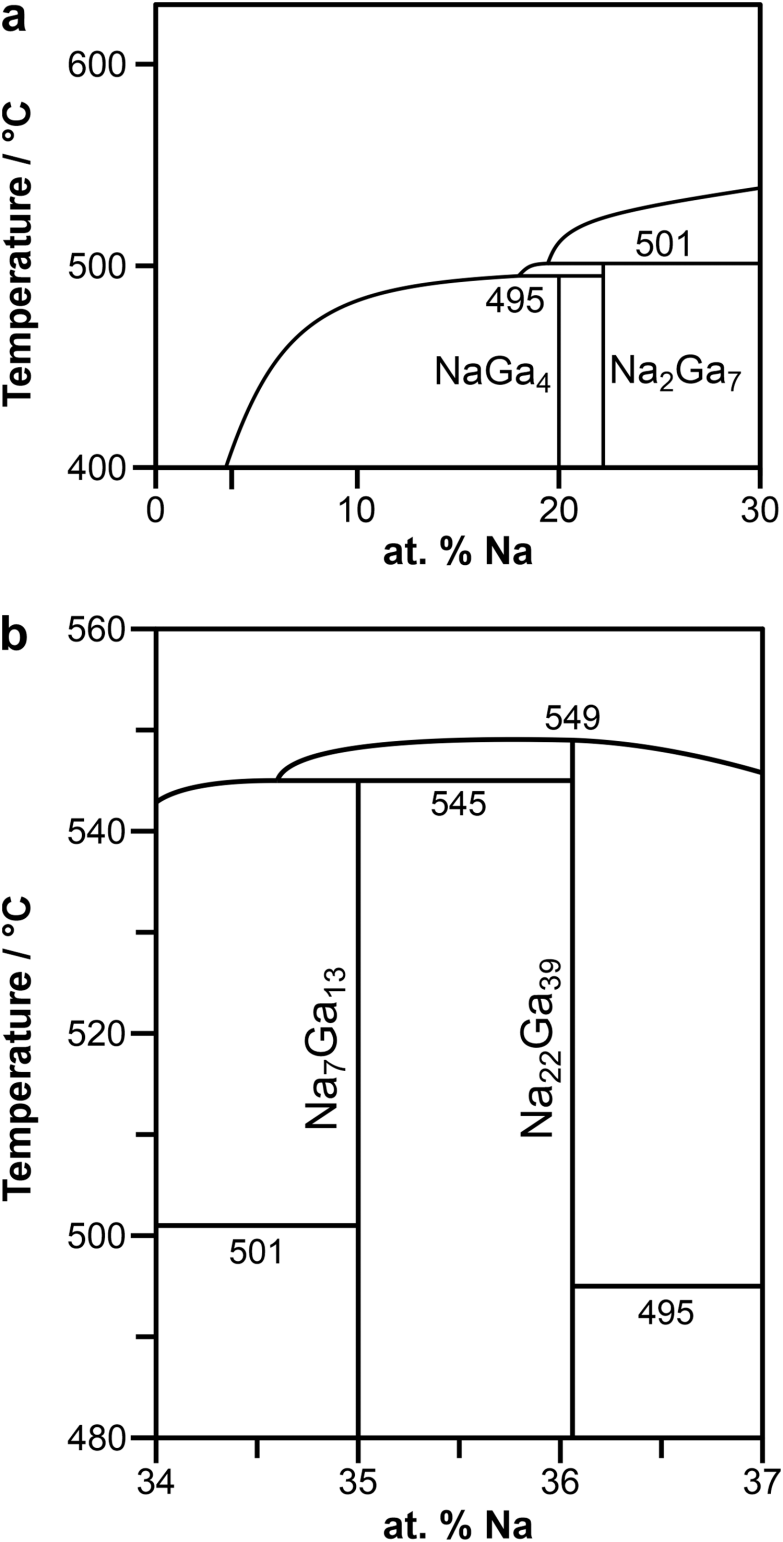
Enlarged sections to clarify the phase relationship between (a) NaGa_4_ and Na_2_Ga_7_ and (b) Na_7_Ga_13_ and Na_22_Ga_39_. The measured temperatures are accurate to 2 °C.

### 3.3 Formation of Na_2_Ga_7_

Single phase samples of Na_2_Ga_7_ (22.2 atom % Na) showed a strong thermal effect at 501(2) °C with a shoulder at approximately 510 °C (Figure S4). The decomposition temperature is therefore only slightly higher than that of NaGa_4_. However, MR-DSC measurements unambiguously confirmed that NaGa_4_ melts prior to Na_2_Ga_7_. Due to the small difference of 6 °C between the peritectic decompositions of NaGa_4_ and Na_2_Ga_7_, the liquidus field of Na_2_Ga_7_ is very narrow. Consequently, when a stoichiometric melt is rapidly cooled to room temperature within 2 min, the PXRD patterns only show reflections of NaGa_4_ and Na_7_Ga_13_. This observation is consistent with the incongruent formation of Na_2_Ga_7_ and its kinetically hindered crystallization. This kinetic inhibition likely explains why the existence of Na_2_Ga_7_ was overlooked in the system until recently.^7^ To probe for a potential homogeneity range of Na_2_Ga_7_, the lattice parameters of the phase were determined from three different compositions annealed between 200–450 °C: a single-phase sample, an equilibrium mixture with NaGa_4_, and an equilibrium mixture with Na_7_Ga_13_. The lattice parameters were found to be constant within experimental error for all samples (for detailed values, see Table 2 in ref 7), confirming that Na_2_Ga_7_ is a line compound. The assignment of Na_2_Ga_7_ as a thermodynamically stable phase has been further corroborated by its presence in the related ternary systems with Na–Li–Ga^13^ and Na–In–Ga.^14^ We conclude that Na_2_Ga_7_ forms peritectically at 501(2) °C from Na_7_Ga_13_ and the corresponding melt (Figure 2a).

### 3.4 Formation of Na_7_Ga_13_ and Na_22_Ga_39_

The coexistence of the phases Na_7_Ga_13_ (35 atom % Na) Na_22_Ga_39_ (36 atom % Na) has been questioned in previous investigations due to their similar stoichiometries.^2,3^ However, the two phases crystallize in different space groups with distinct crystal structures.^5,8,9^ In agreement with Itami et al.,^1^ we confirm the equilibrium coexistence of both phases by PXRD. Single-phase samples of each compound were obtained by annealing stoichiometric mixtures at 450 °C in sealed Ta ampules (Figure 3a). Crucially, a sample with an intermediate composition, prepared under identical conditions, resulted in a 1:1 mixture of both phases, as evidenced by the presence of reflections from both Na_7_Ga_13_ and Na_22_Ga_39_ in the PXRD pattern (Figure 3b). This observation unambiguously confirms the existence of a two-phase region in the phase diagram. Both phases coexist after further annealing at 300 and 200 °C. Consistently, Cordier et al. reported that Na_7_Ga_13_ forms with a Ga excess, while Na_22_Ga_39_ (denoted as Na_7_Ga_13_–II in their work) forms with a Na excess.^9^ Determining the precise thermodynamic relationship between Na_7_Ga_13_ and Na_22_Ga_39_ proved challenging. HF-DSC measurements on single-phase samples in closed ampules showed indistinguishable onset temperatures of 549(2) °C, with no further thermal effects observed (Figure S5a,c). This effect was also described by Itami et al.^1^ However, a Na_7_Ga_13_ sample in an open crucible (implying a small Na loss) in a DTA/TG experiment revealed a lower onset temperature of 545(2) °C (Figure S5b, ref 7). The lower decomposition temperature for Na_7_Ga_13_ was further confirmed by MR-DSC measurements of Na_7_Ga_13_ and Na_22_Ga_39_, which consistently showed an onset at 545(2) °C. While MR-DSC measurements may not provide highly reliable absolute reaction temperatures, in these specific measurements, the baseline was ideally flat (Figure S5d,e). More importantly, the relative measurements indicate that Na_7_Ga_13_ melts slightly but definitively lower than Na_22_Ga_39_. In addition, an MR-DSC experiment on an intermediate composition (35.8 atom % Na), using Na_7_Ga_13_ as a reference, showed no depression of the melting point (Figure S5f). In a eutectic system of Na_7_Ga_13_ and Na_22_Ga_39_, the intermediate composition would be expected to melt first. Taken together, these results point toward a peritectic reaction Na_7_Ga_13_ → *L* + Na_22_Ga_39_ at 545(2) °C and a dystectic reaction Na_22_Ga_39_ → *L* at *T* = 549(2) °C (Figures 1 and 2b). In conclusion, Na_7_Ga_13_ and Na_22_Ga_39_ are thermodynamically distinct phases with minimally different stoichiometries and no detectable homogeneity ranges.

**Figure 3:**
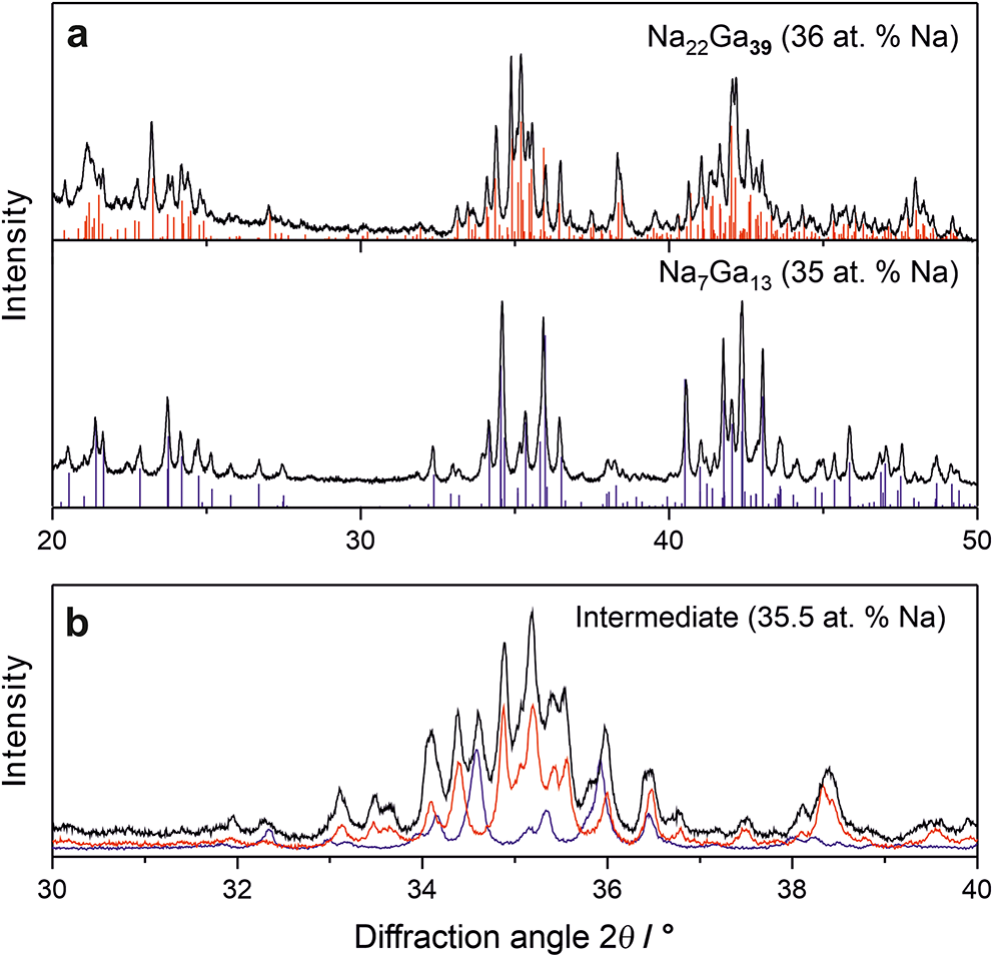
Powder X-ray diffraction patterns of the Na–Ga system. (a) Na_7_Ga_13_ and Na_22_Ga_39_ obtained from annealing stoichiometric mixtures at 450 °C for 1 week. Calculated intensities are based on single-crystal structure data.^5,8^ (b) A sample from the two-phase region (35.5 atom % Na) reveals reflections of both Na_7_Ga_13_ (blue) and Na_22_Ga_39_ (red), confirming their equilibrium coexistence at 450 °C.

### 3.5 Monotectic Region

Na_22_Ga_39_ is the most sodium-rich binary compound in the Na–Ga system. Given its congruent melting behavior, the peritectic isotherm *L* + Na_22_Ga_39_ → Na_7_Ga_13_ proposed by Itami et al.^1^ can be ruled out. Instead, our results confirm the existence of a monotectic reaction isotherm reported by Pelton et al.^2^ and Okamoto^3^ (Figure 1). However, in our experiments, the invariant monotectic temperature of 495(2) °C was determined (Figure S6), which is significantly lower than the previously published values of 519 ^2^ and 524 °C.^3^ The critical temperature of the liquid miscibility gap was estimated to be 523(2) °C for the composition of 75 atom % Na. This value is closer to the estimate by Pelton et al.,^2^ while the significantly higher critical temperature of 728 °C suggested by Okamoto^3^ and Wang et al.^15^ is not supported by our data. A comprehensive mapping of the extent of the two-liquid region was beyond the scope of this study.

### 3.6 Na-Rich Samples (>90 Atom % Na)

The solubility of gallium in sodium is vanishingly small, with a reported value of 0.2 ppm Ga in liquid Na at 98 °C.^11^ The solubility in the solid state can be assumed to be even lower. Consequently, any depression of the eutectic temperature relative to the melting point of pure sodium is expected to be significantly less than 0.1 °C. Detection of such a small thermal effect is beyond the resolution of the methods employed in this study. The thermal effects observed in our measurements consistently occurred at 98(2) °C, which corresponds to the melting point of pure sodium (97.8 °C) and suggests the presence of a degenerate eutectic.

## 4 Conclusion

In this work, the phase equilibria in the Na–Ga system were reinvestigated, yielding a revised phase diagram that resolves longstanding discrepancies in the literature. The study confirms the existence of the four binary phases NaGa_4_, Na_2_Ga_7_, Na_7_Ga_13_, and Na_22_Ga_39_. We find that Na_22_Ga_39_ is the sodium-richest and congruently melting phase in the system, while Na_7_Ga_13_, Na_2_Ga_7_, and NaGa_4_ all form peritectically (Table 1). None of these phases exhibit a measurable homogeneity range. Despite their very similar compositions, Na_7_Ga_13_, and Na_22_Ga_39_ are neighboring phases with distinct crystal structures. Furthermore, the presence of a monotectic reaction in the sodium-rich region, forming Na_22_Ga_39_ and a melt, was confirmed, albeit at a lower temperature than previously suggested. The thermodynamic model is obtained from complementary HF-DSC and PXRD data. The utility of mutual reference DSC for deconvoluting closely spaced thermal events is demonstrated.

**Table 1:** Phases and Formation Reactions in the Binary System Na–Ga

compound	atom % Na	space group	formation (cooling)	temp. (°C)
NaGa_4_	20	*I*4/*mmm*	*L* + Na_2_Ga_7_ → NaGa_4_ peritectic	495(2)
Na_2_Ga_7_	22	*Pnma*	*L* + Na_7_Ga_13_ → Na_2_Ga_7_ peritectic	501(2)
Na_7_Ga_13_	35	*R*3̅*m*	*L* + Na_22_Ga_39_ → Na_7_Ga_13_ peritectic	545(2)
Na_22_Ga_39_	36	*Pnma*	*L* → Na_22_Ga_39_ congruent	549(2)

## Supplementary Material



## Notes

HF-DSC plots (all samples); MR-DSC plots (Na_7_Ga_13_ and Na_22_Ga_39_); DTA/TG measurement (Na_7_Ga_13_) (PDF)
