# Supplementary material for: Investigation of the Na–Ga Phase Diagram
Source: ACS Omega. 2026 Jan 5;11(2):3593–7. doi: 10.1021/acsomega.5c11531 (PMC12824930; doi:10.1021/acsomega.5c11531)
Supplement: Supplementary file 1 [file ao5c11531_si_001.pdf]

Investigation of the Na-Ga Phase Diagram

Chia-Chi Yu, Marcus Schmidt, Michael Baitinger, Yuri Grin.*

Max-Planck-Institut für Chemische Physik fester Stoffe, Nöthnitzer Straße 40, 01187 Dresden

S1a. HF-DSC measurement of elemental **Ga**, heating curve. **0 % Na**

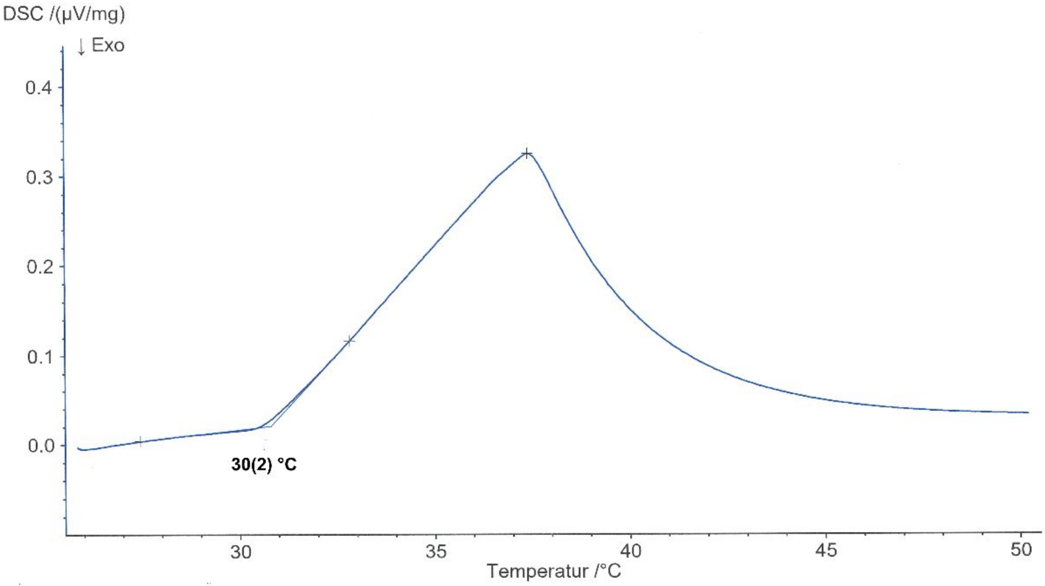

crucible:	Nb, welded	Measured range:	25°C-50°C
sample mass:	56 mg	Heating rate:	2 K/min

S1b. HF-DSC measurement of Na_2Ga_{98}, heating curve. **2 % Na**

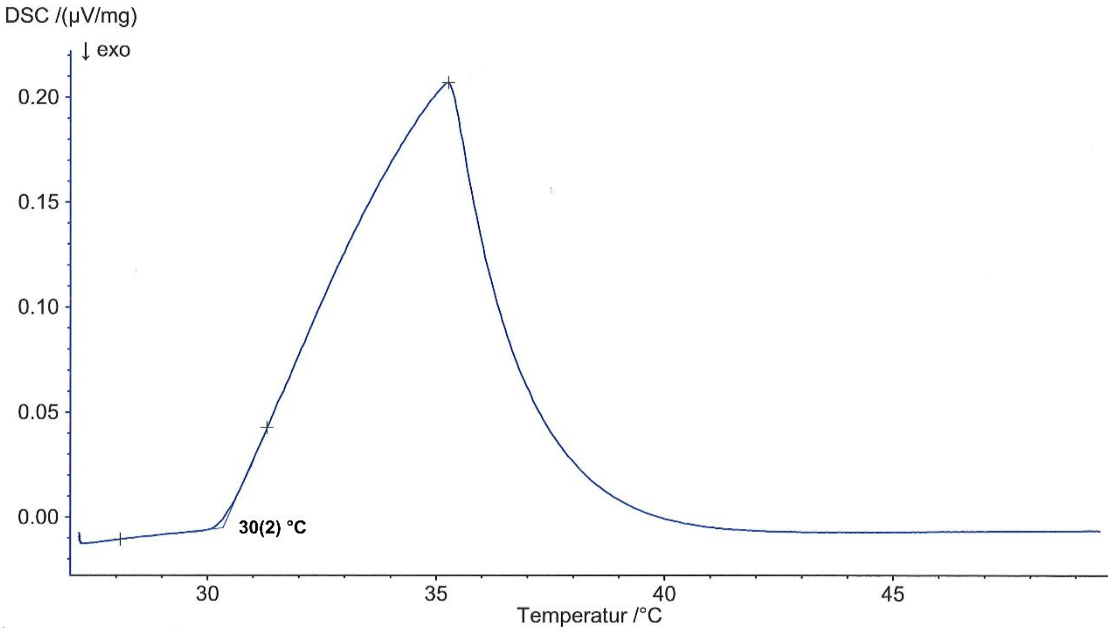

crucible:	Nb, welded	Measured range:	25°C-50°C
sample mass:	55 mg	Heating rate:	1 K/min

S1c. HF-DSC measurement of Na_4Ga_{96}, heating curve. **4 % Na**

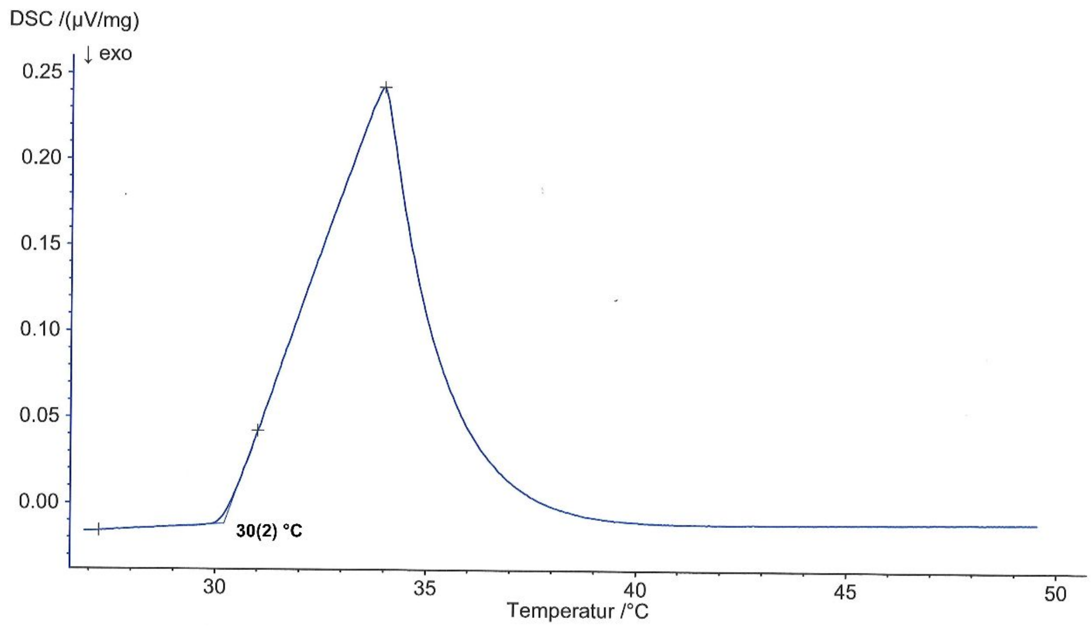

crucible:	Nb, welded	Measured range:	25°C-50°C
sample mass:	41 mg	Heating rate:	1 K/min

S2a. HF-DSC measurement of Na_9Ga_{91}, heating curve. **9 % Na**

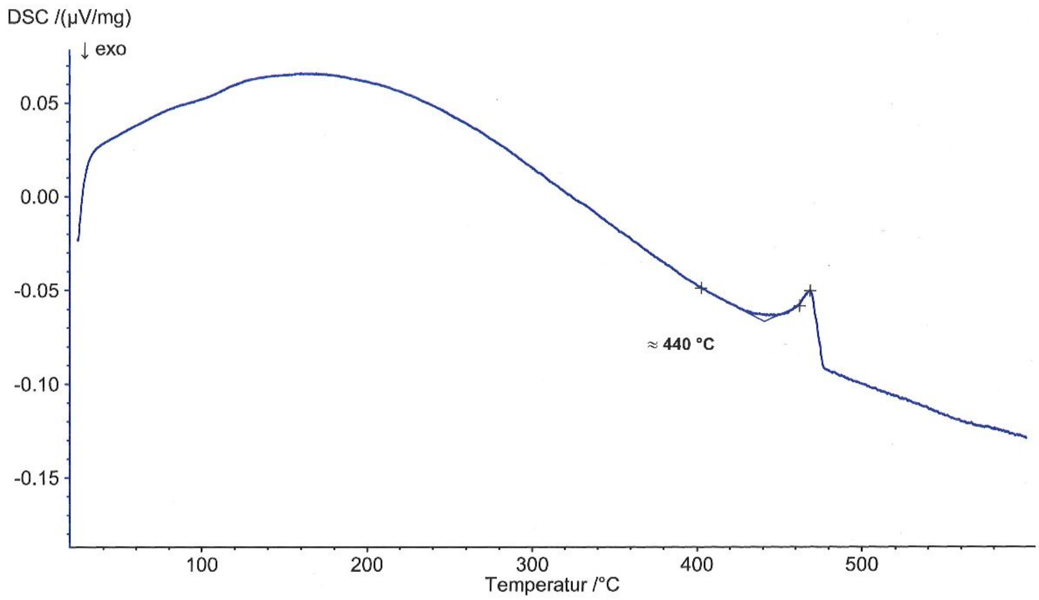

crucible:	Nb, welded	Measured range:	25°C-600°C
sample mass:	25 mg	Heating rate:	2 K/min

S2b. HF-DSC measurement of $Na_{10}Ga_{90}$, heating curve. **10 % Na**

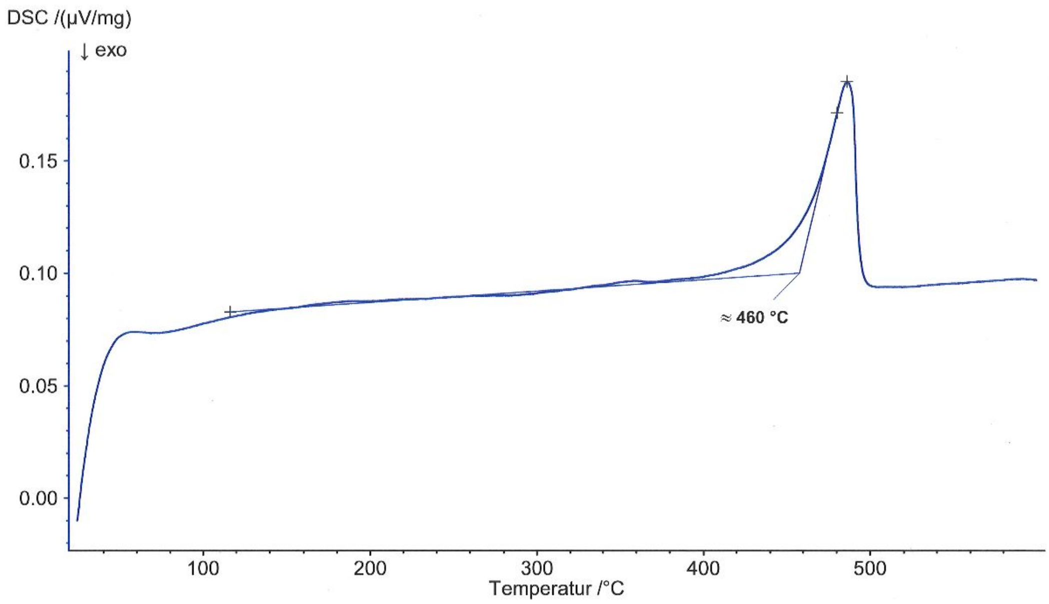

crucible:	Nb, welded	Measured range:	25°C-600°C
sample mass:	61 mg	Heating rate:	5 K/min

S2c. HF-DSC measurement of $Na_{11}Ga_{89}$, heating curve. **11 % Na**

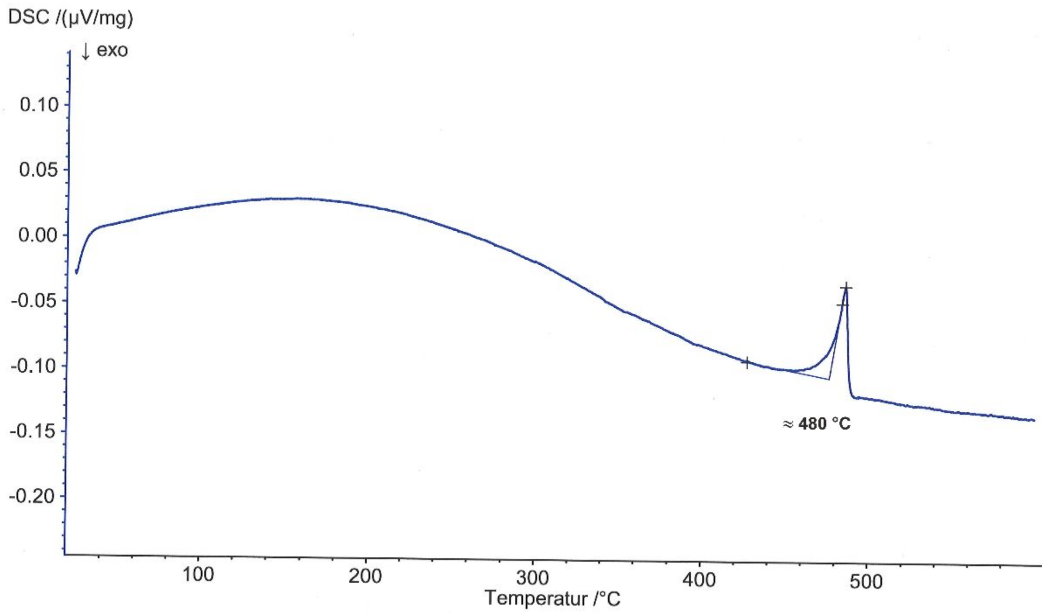

crucible:	Nb, welded	Measured range:	25°C-600°C
sample mass:	25 mg	Heating rate:	2 K/min

S3a. HF-DSC measurement of $Na_{18}Ga_{82}$, heating curve. **18 % Na**

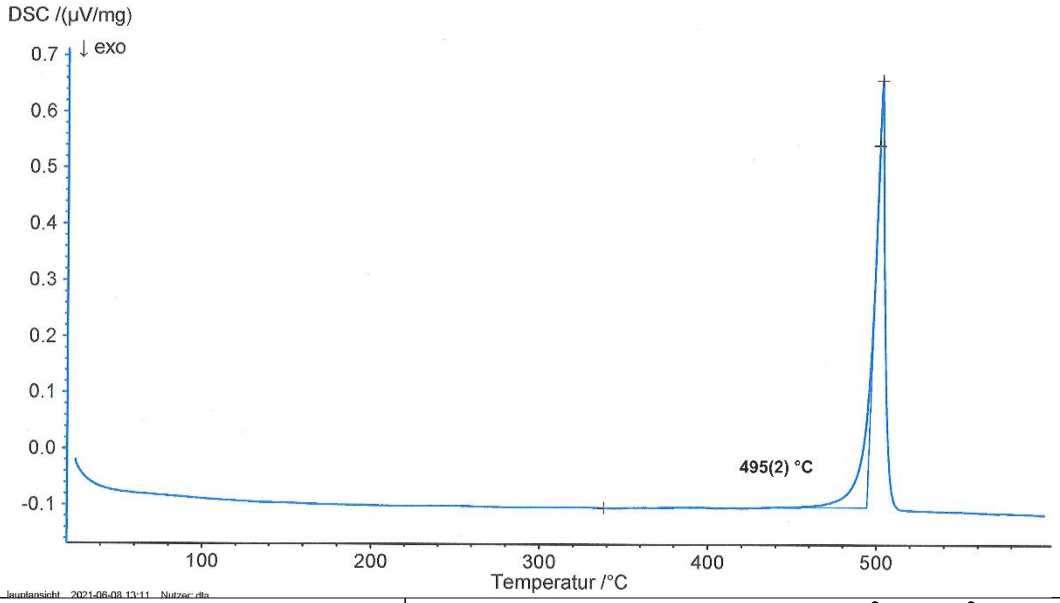

crucible:	Nb, welded	Measured range:	25°C-600°C
sample mass:	31 mg	Heating rate:	5 K/min

S3b. HF-DSC measurement of $Na_{20}Ga_{80}$ (**$NaGa_4$**), heating curve. **20 % Na**

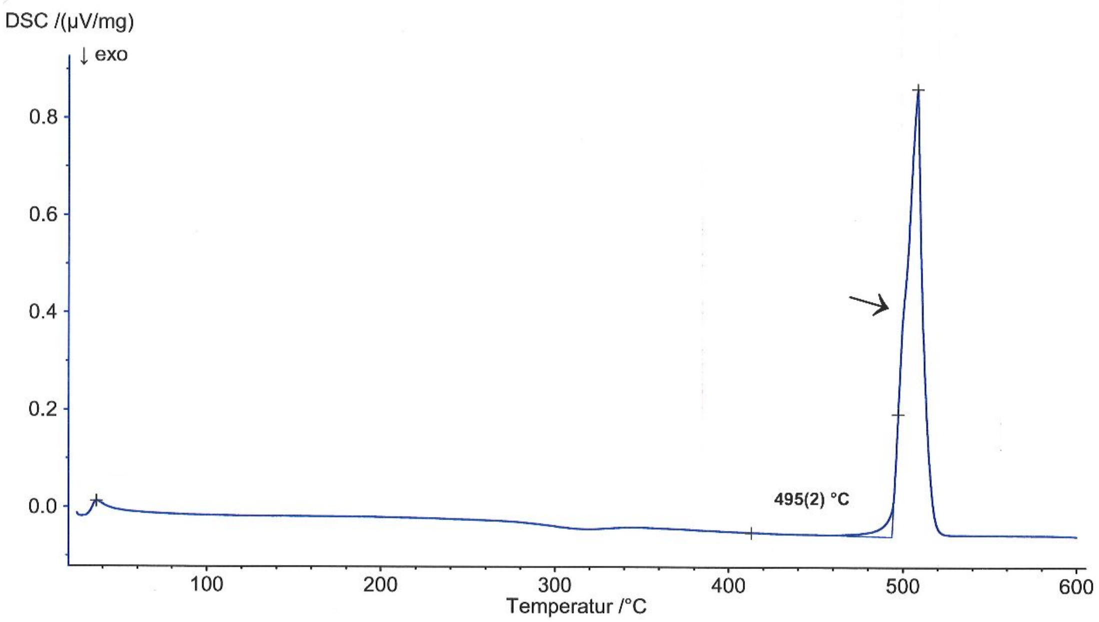

crucible:	Nb, welded	Measured range:	25°C-600°C
sample mass:	52 mg	Heating rate:	5 K/min

**S3c.** HF-DSC measurement of $Na_{21}Ga_{79}$, heating curve.			**21 % Na**

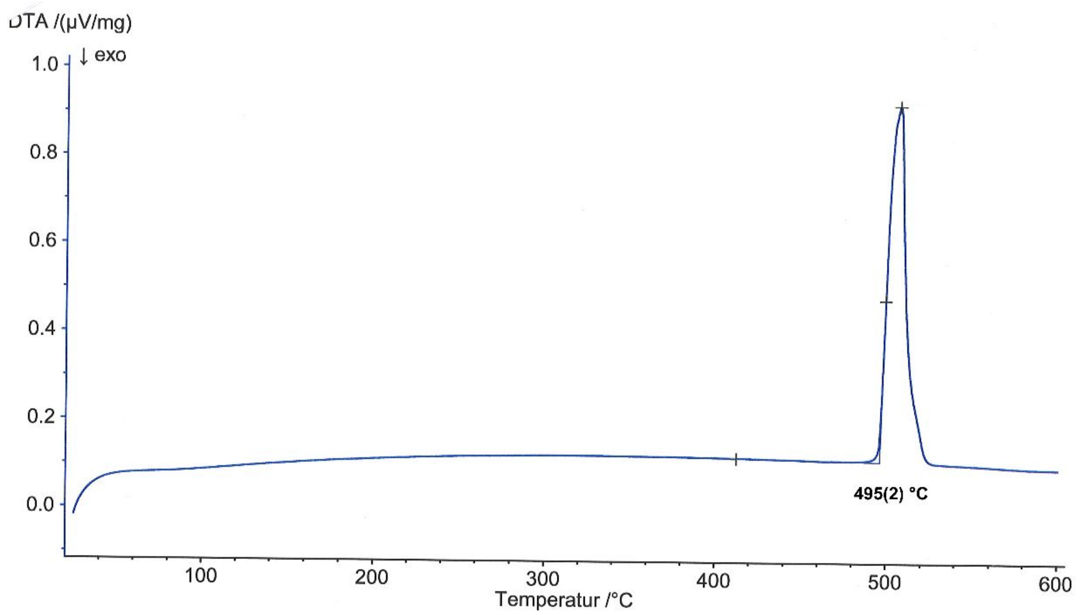

crucible:	Nb, welded	Measured range:	25°C-600°C
sample mass:	31 mg	Heating rate:	5 K/min

**S4.** HF-DSC measurement of $Na_{22.2}Ga_{77.8}$ (**Na_2Ga_7**), heating curve.			**22.2 % Na**

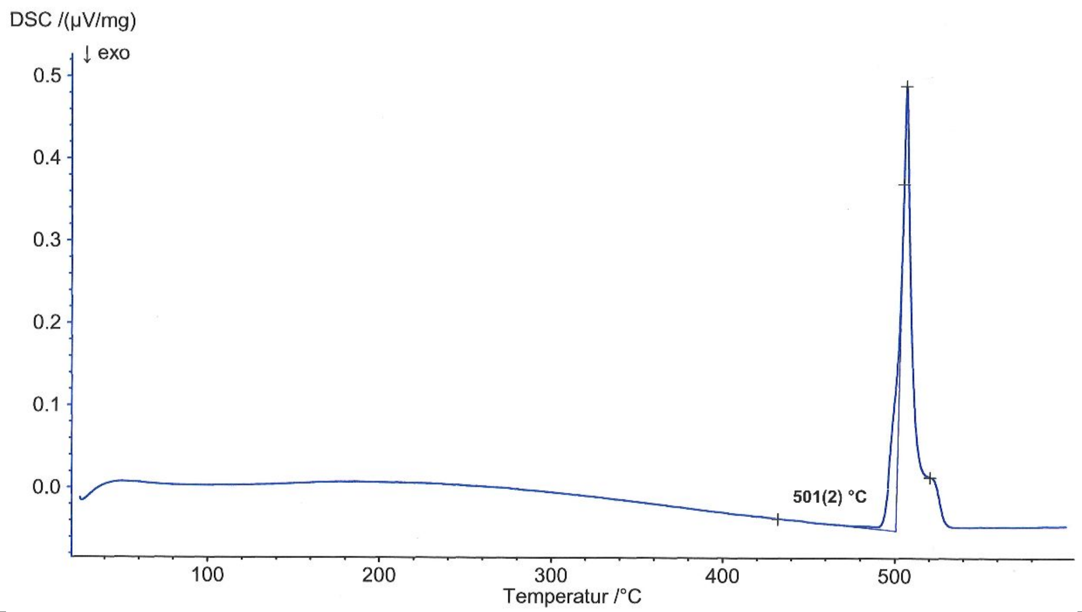

crucible:	Nb, welded	Measured range:	25°C-600°C
sample mass:	50 mg	Heating rate:	5 K/min

**S5a.** HF-DSC measurement of $Na_{35}Ga_{65}$ (**Na_7Ga_{13}**), heating curve.			**35 % Na**
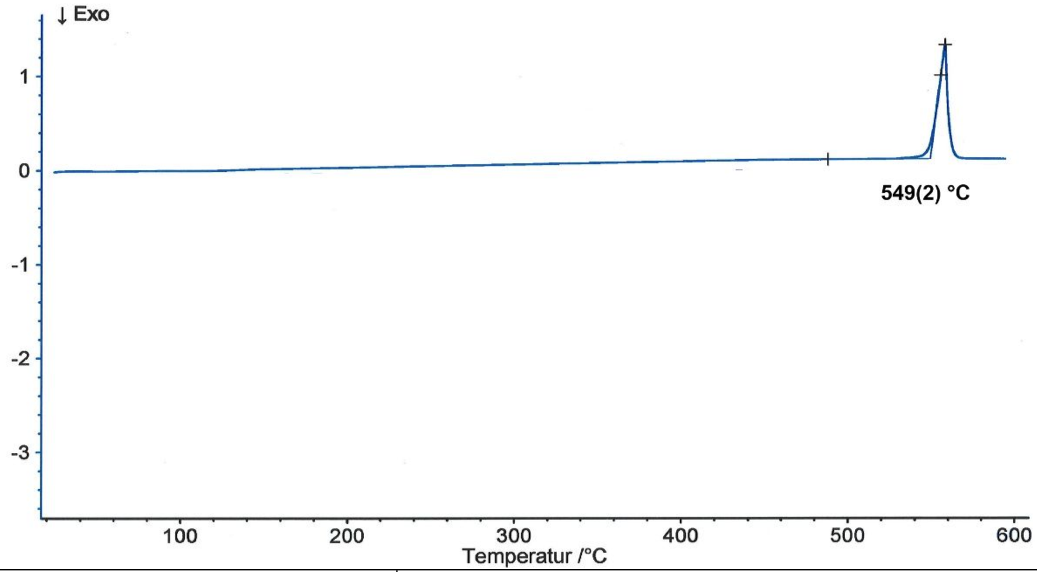			
crucible:	Nb, welded	Measured range:	25°C-600°C
sample mass:	30 mg	Heating rate:	5 K/min

**S5b.** DTA/TG measurement of $Na_{35}Ga_{65}$ (**Na_7Ga_{13}**), heating curve.			**35 % Na**
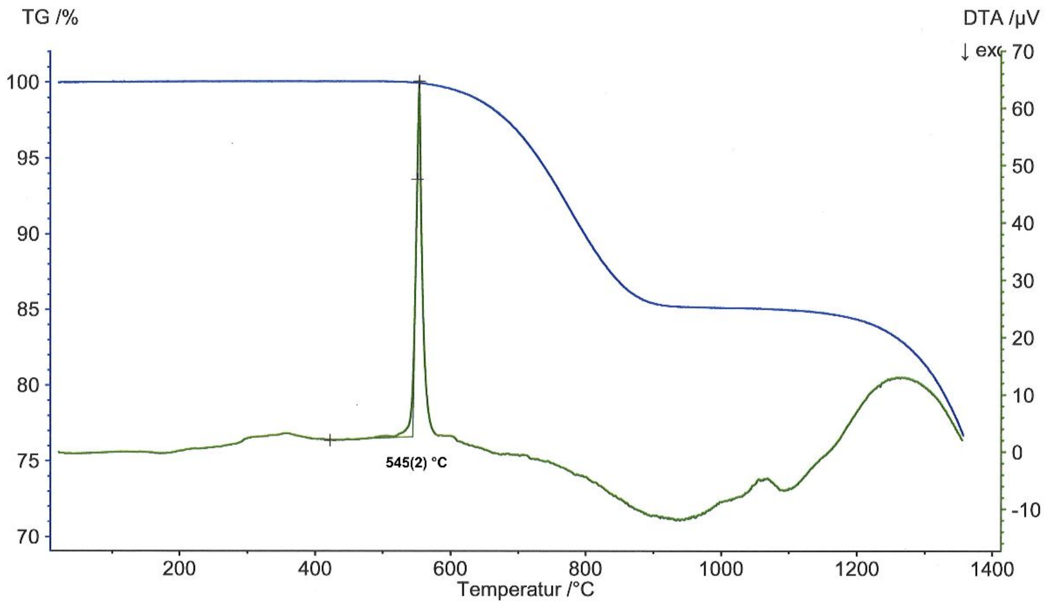			
crucible:	Ta, open	Measured range:	25°C-600°C
sample mass:	30 mg	Heating rate:	10 K/min

**S5c.** HF-DSC measurement of $Na_{36.1}Ga_{63.9}$ (**$Na_{22}Ga_{39}$**), heating curve.			**36 % Na**

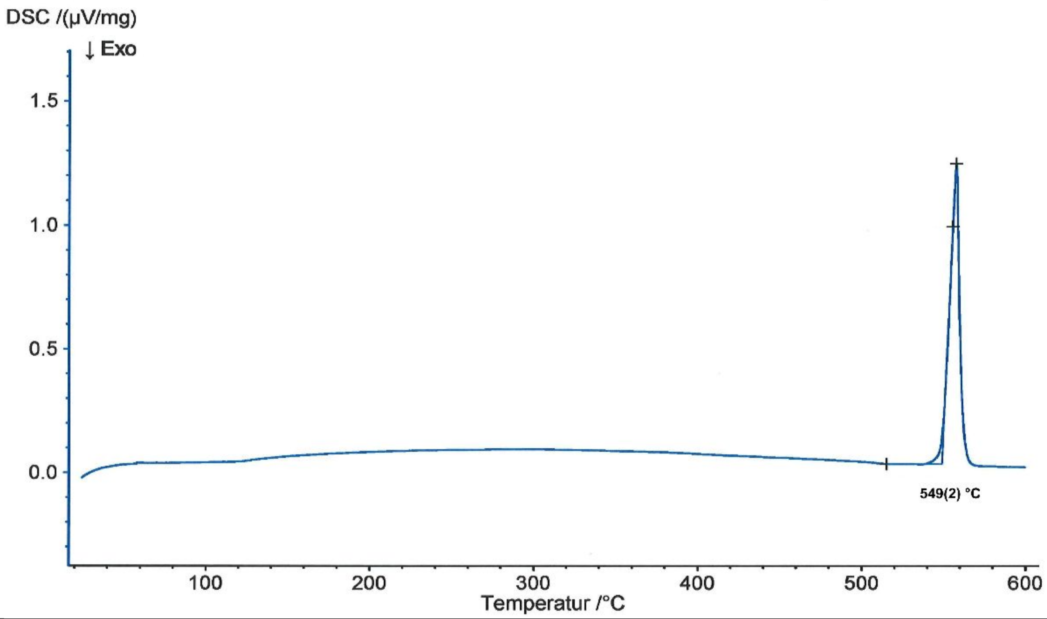

crucible:	Nb, welded	Measured range:	25°C-600°C
sample mass:	31 mg	Heating rate:	5 K/min

**S5d.** MR-DSC measurement, heating curve.		**35 % Na**
Sample position: **$Na_{22}Ga_{39}$** (2)	Reference position: **Na_7Ga_{13}** (1)	**36 % Na**

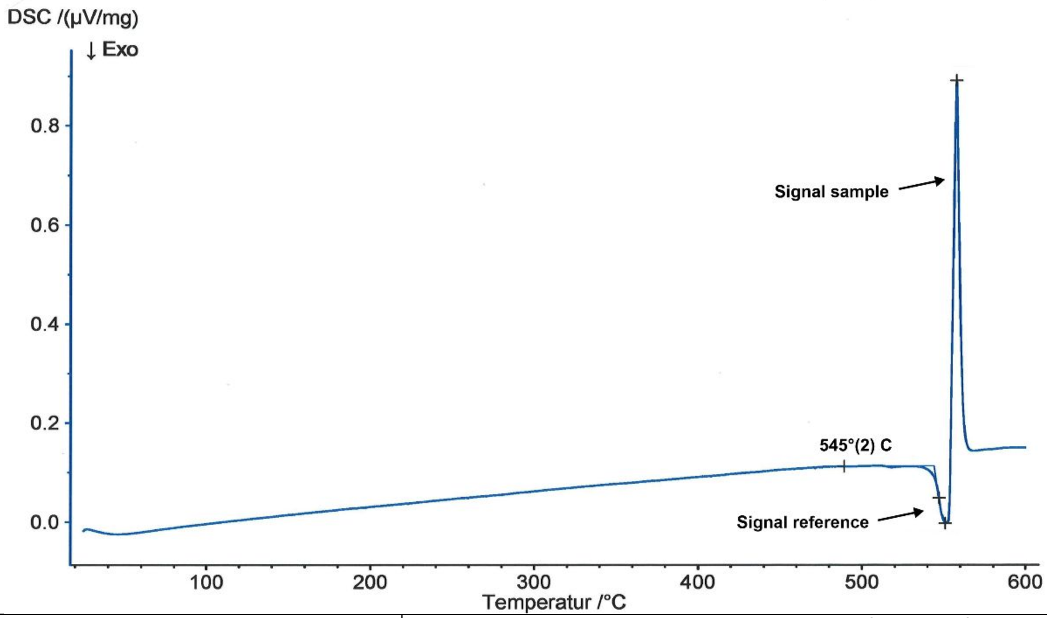

crucible:	Nb, welded	Measured range:	25°C-600°C
sample mass:	35 mg	Heating rate:	5 K/min

**S5e.** MR-DSC measurement, heating curve.		**35 % Na**
Sample position: **Na_7Ga_{13}** (1)	Reference position: **$Na_{22}Ga_{39}$** (2)	**36 % Na**

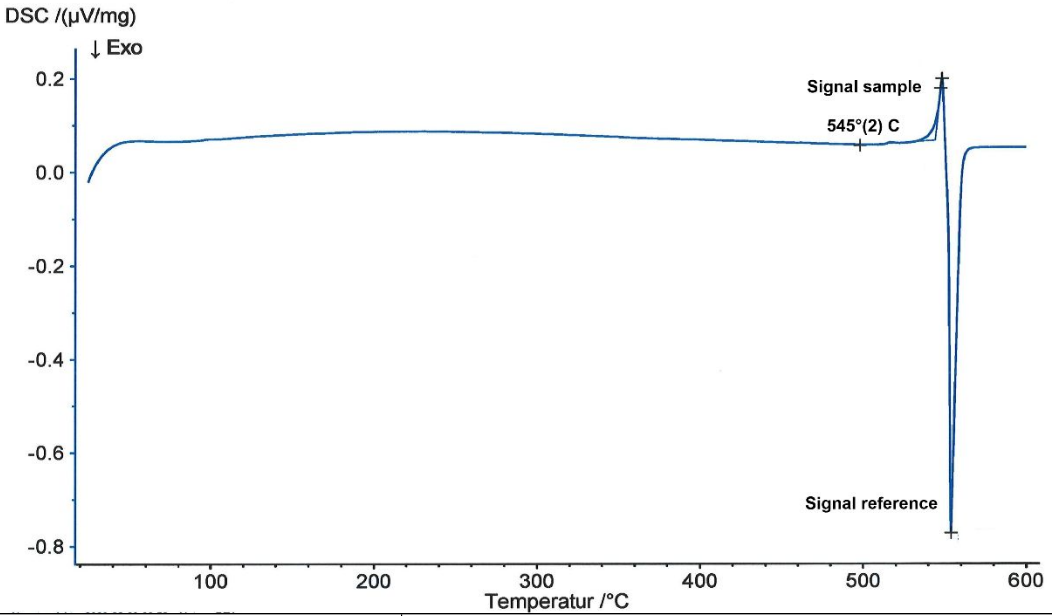

crucible:	Nb, welded	Measured range:	25°C-600°C
sample mass:	31 mg	Heating rate:	5 K/min

**S5f.** MR-DSC measurement, heating curve.		**35 % Na**
Sample position: **Na_7Ga_{13}** (1)	Reference position: **$Na_{35.8}Ga_{64.2}$**	**35.8 % Na**

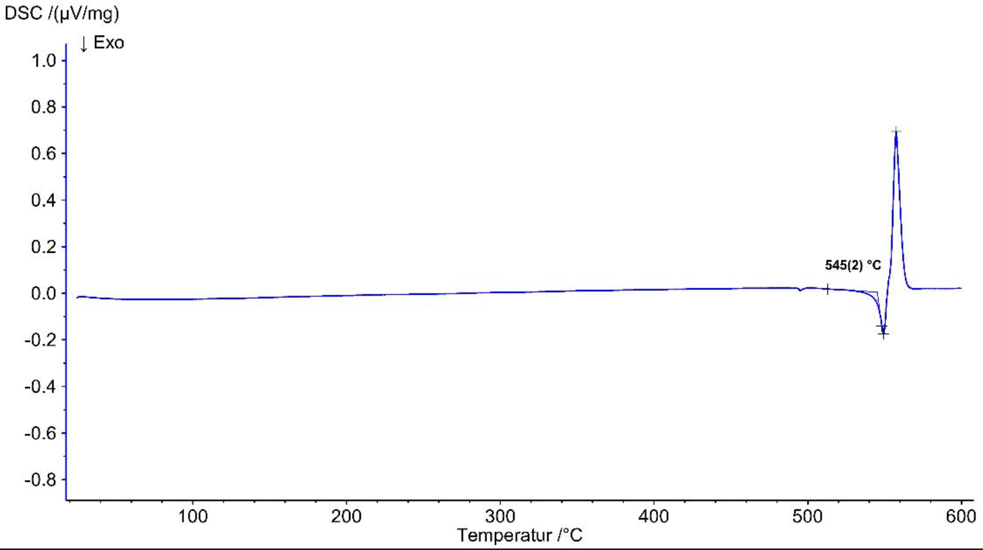

crucible:	Nb, welded	Measured range:	25°C-600°C
sample mass:	33 mg	Heating rate:	5 K/min

S6a. HF-DSC measurement of $Na_{60}Ga_{40}$, heating curve. **60 % Na**

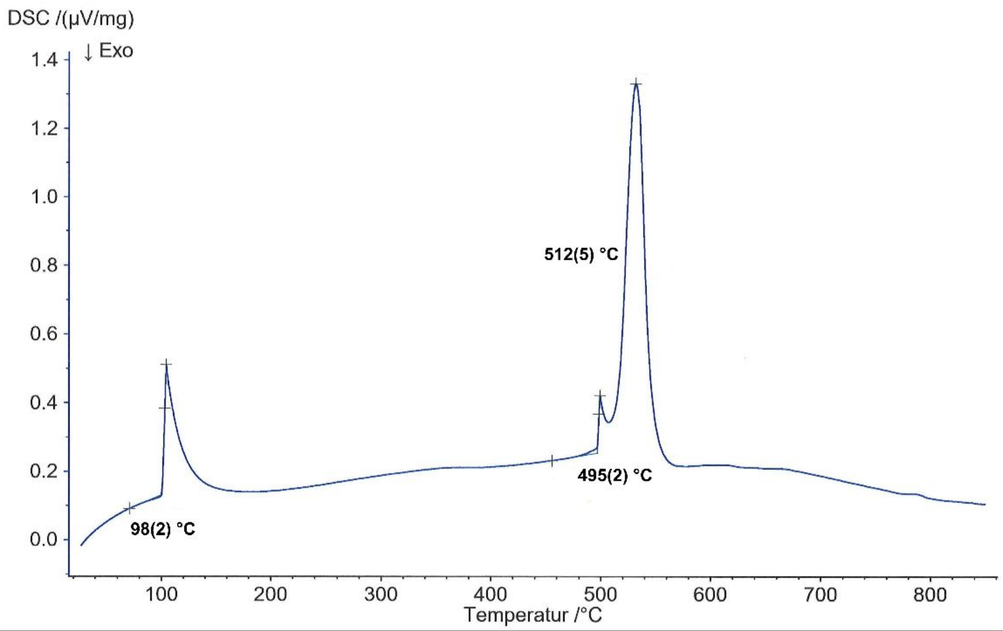

crucible:	steel, screw cap	Measured range:	25°C-600°C
sample mass:	48 mg	Heating rate:	10 K/min

S6b. HF-DSC measurement of $Na_{75}Ga_{25}$, heating curve. **75% Na**

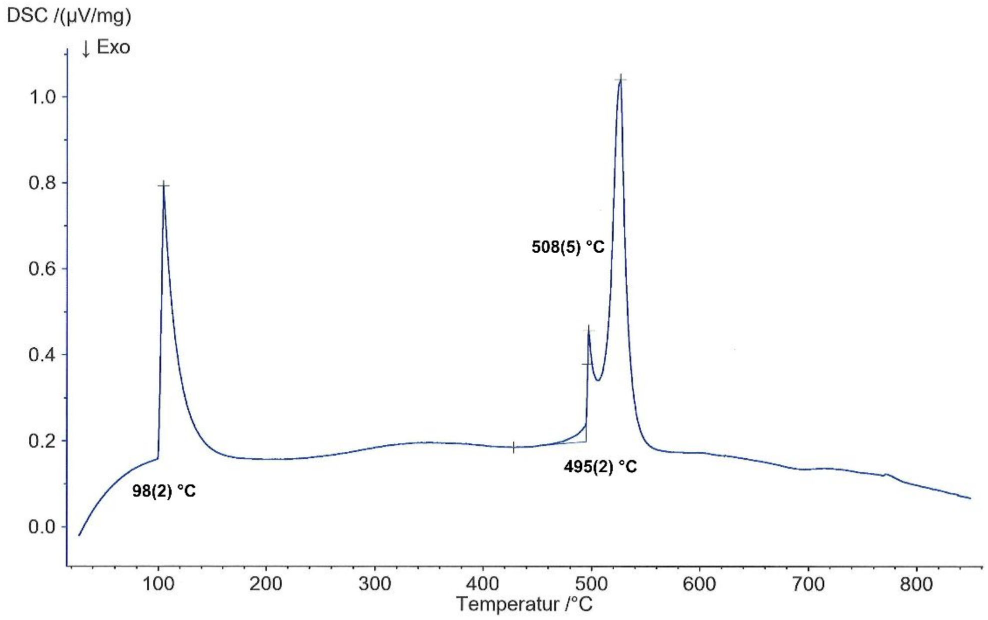

crucible:	steel, screw cap	Measured range:	25°C-600°C
sample mass:	35 mg	Heating rate:	10 K/min

S6c. HF-DSC measurement of $Na_{80}Ga_{20}$, heating curve. **80% Na**

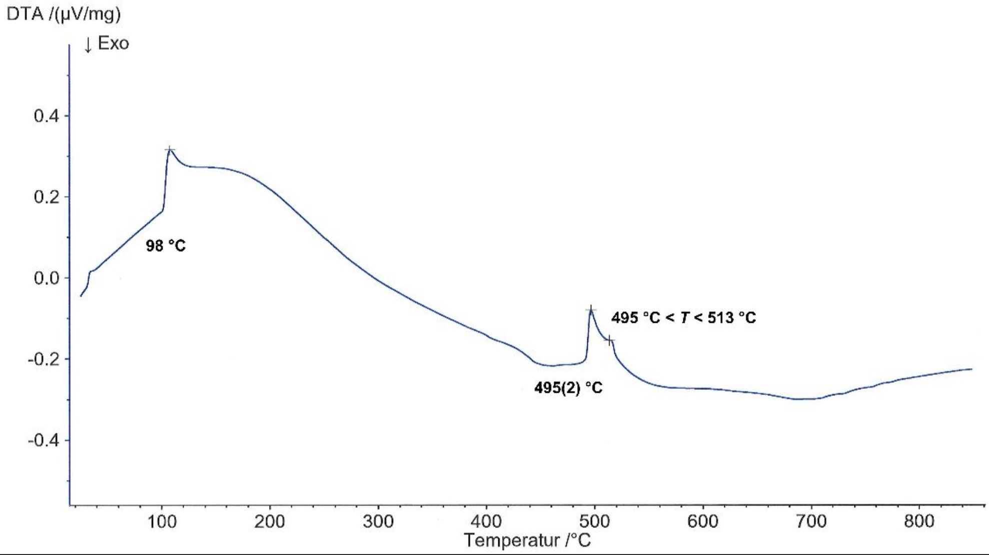

crucible:	steel, screw cap	Measured range:	25°C-600°C
sample mass:	45 mg	Heating rate:	10 K/min

S6d. HF-DSC measurement of $Na_{80}Ga_{205}$, heating curve. **80% Na**

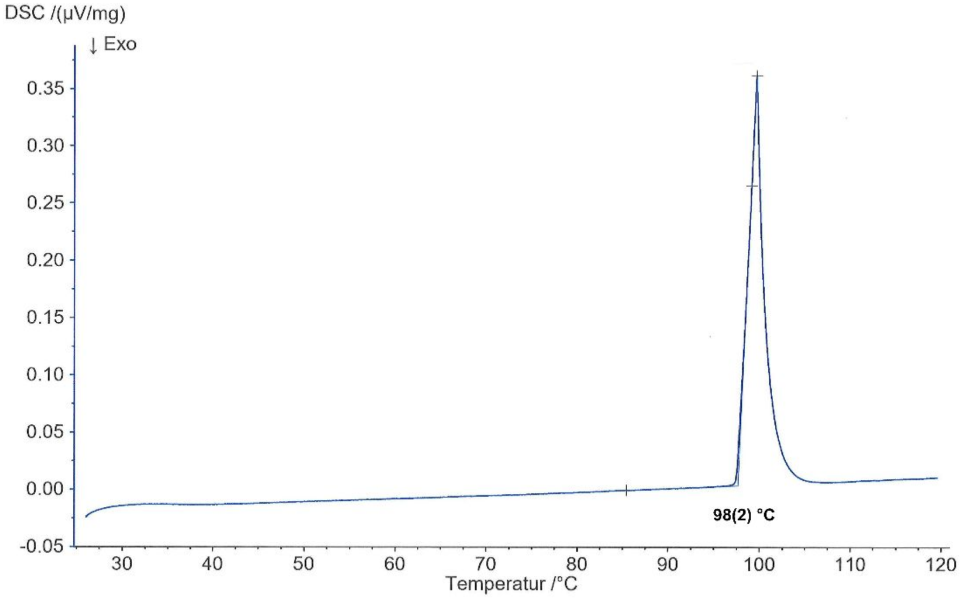

crucible:	steel, screw cap	Measured range:	25°C-120°C
sample mass:	28 mg	Heating rate:	1 K/min

**S7a.** HF-DSC measurement of $Na_{95}Ga_5$, heating curve.		**95% Na**	

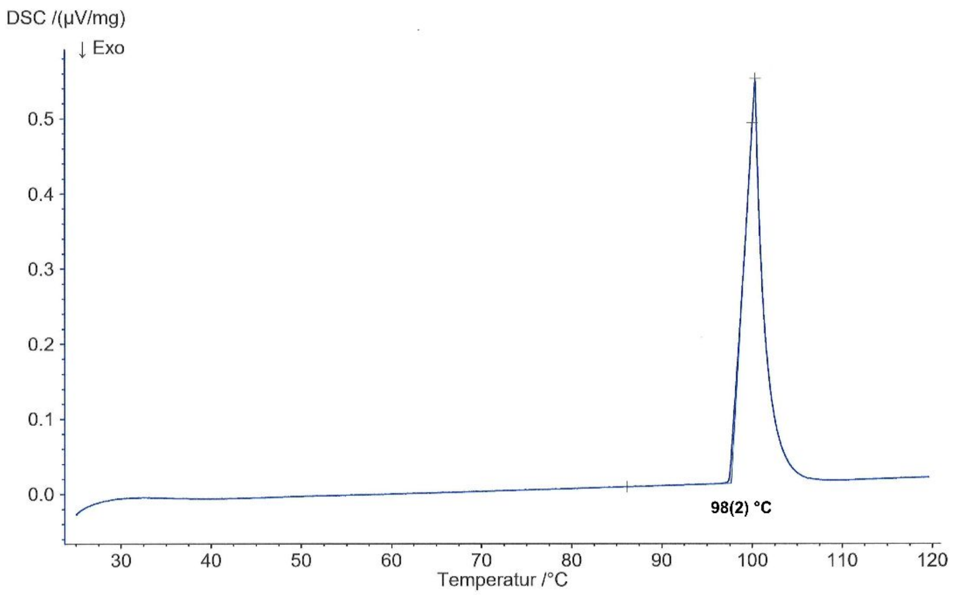

crucible:	steel, screw cap	Measured range:	25°C-120°C
sample mass:	25 mg	Heating rate:	1 K/min

**S7b.** HF-DSC measurement of elemental Na, heating curve.		**100% Na**	

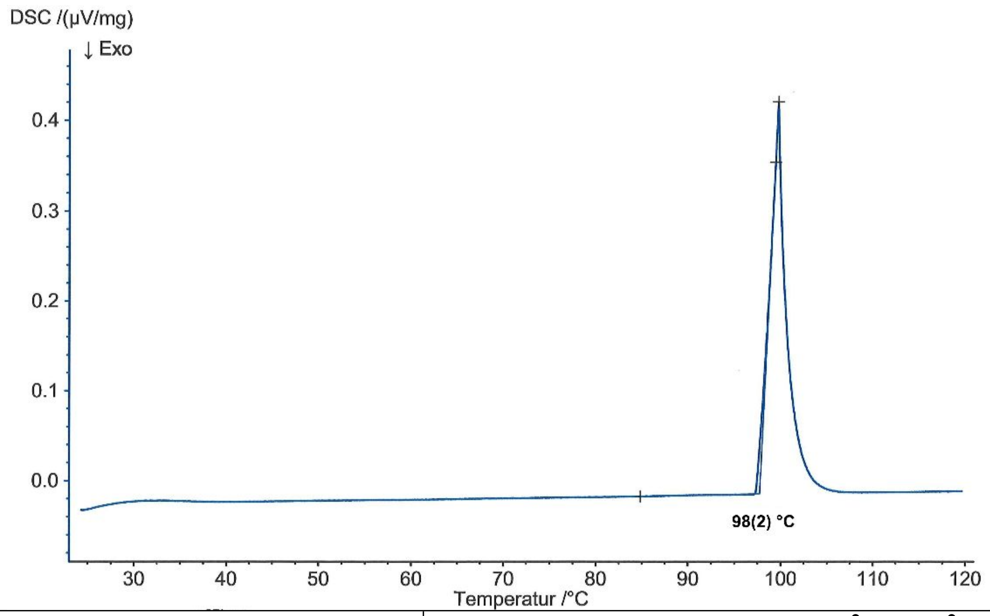

crucible:	steel, screw cap	Measured range:	25°C-120°C
sample mass:	20 mg	Heating rate:	1 K/min
